# Indian Psychiatric epidemiological studies: Learning from the past

**DOI:** 10.4103/0019-5545.69220

**Published:** 2010-01

**Authors:** Suresh Bada Math, Ravindra Srinivasaraju

**Affiliations:** Department of Psychiatry, National Institute of Mental Health and Neuro Sciences (Deemed University), Bangalore - 560 029, India

**Keywords:** Psychiatric Epidemiology, Community, Prevalence, Research

## Abstract

The objective of this paper is to provide a systematic review on the epidemiology of psychiatric disorders in India based on the data published from 1960 to 2009. Extensive search of PubMed, NeuroMed, Indian Journal of Psychiatry website and MEDLARS using search terms “psychiatry” “prevalence”, “community”, and “epidemiology” was done along with the manual search of journals and cross-references. Retrieved articles were systematically selected using specific inclusion and exclusion criteria. Epidemiological studies report prevalence rates for psychiatric disorders varying from 9.5 to 370/1000 population in India. These varying prevalence rates of mental disorders are not only specific to Indian studies but are also seen in international studies. Despite variations in the design of studies, available data from the Indian studies suggests that about 20% of the adult population in the community is affected with one or the other psychiatric disorder. Mental healthcare priorities need to be shifted from psychotic disorders to common mental disorders and from mental hospitals to primary health centers. Increase in invisible mental problems such as suicidal attempts, aggression and violence, widespread use of substances, increasing marital discord and divorce rates emphasize on the need to prioritize and make a paradigm shift in the strategies to promote and provide appropriate mental health services in the community. Future epidemiological research need to focus on the general population from longitudinal prospective involving multi-centers with assessment of disability, co-morbidity, functioning, family burden and quality of life.

## INTRODUCTION

Psychiatric epidemiology is the study of the distribution and determinants of mental illness frequency in human beings, with the fundamental aim of understanding and controlling the occurrence of mental illness. Psychiatric epidemiology deals with important components such as disease/disorder, distribution and frequency of disease/disorder, determinants of disease/disorder, human population and methods employed to control the occurrence of illness.[1]

Mental disorders constitute a wide spectrum ranging from sub-clinical states to very severe forms of disorders. Mental health problems can attain the disorder/disease/syndrome level, which are usually considered easy to recognize, define, diagnose and treat. Hence, they can be called, ‘*Visible Mental Health Problems*’ in a community. These visible mental health problems are again classified into *Major mental disorders* and *Minor mental disorders*. Major mental disorders are easy to recognize and commonly seen in mental hospitals, however, minor mental disorders are common in the community. Another group of mental health problems remains at the sub-clinical/non-clinical/sub-syndromal level and are usually related to the behavior of an individual. They are difficult to recognize, define and diagnose. Hence, they are called, ‘*Invisible Mental Health Problems*’. Psychiatric epidemiological studies have ignored this category because of the difficulty in defining and identifying the case. It has also been argued by many researchers, not to pathologize the problems faced by the individuals.

**Figure 1:**
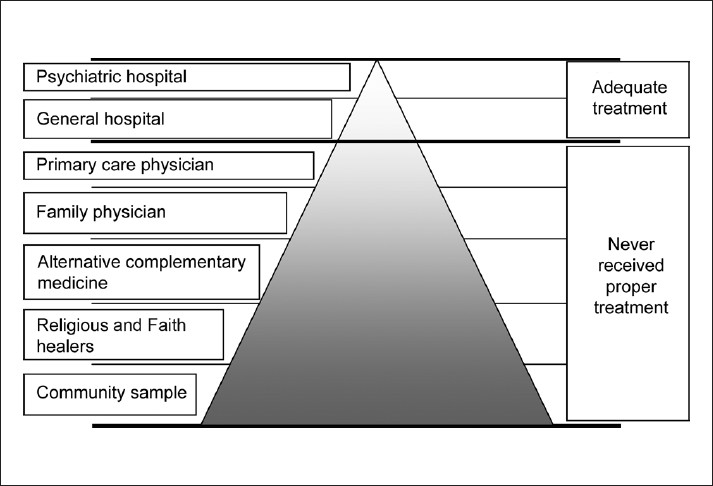
Pathways to mental health care pyramid in developing countries

Popular approaches to measure the disease frequency in a given population are, (i) hospital catchment population approach and (ii) community survey.[2] Hospital-based approach counts the number of cases diagnosed by a clinician (as numerator) and the catchment population served by the hospital facilities (as denominator). The generalizability of the findings of these hospital studies to the community setting is very difficult, especially in developing countries because of the following reasons: a) hospital samples generally comprise severely ill patients, b) patients may approach the hospital far away from home (not the nearest treatment center) due to stigma and discrimination and c) disease variants seen in the community may be with mild impairment. This can be explained by the pathways to care pyramid as shown in Figure 1. At the bottom of the pyramid remains a huge population of mentally ill patients who may not receive treatment at all. Hence, to get the true picture, community sampling is advocated.

Psychiatric epidemiology lags behind other branches of epidemiology due to difficulties encountered in conceptualizing, defining a case and diagnosing, sampling technique, lack of trained manpower, poor knowledge, data collection from a single informant, systematic under - reporting, stigma, lack of adequate funding and low priority of mental health in the health policy.[3–4] In spite of the above challenges there have been endeavors into descriptive psychiatric epidemiological studies, but advances with respect to cost-effective, analytical and prospective experimental epidemiological studies have been minimal.

Descriptive epidemiological studies have provided data about the prevalence of mental disorders in the community. However, many researchers have expressed reservations about the comparison of various epidemiological studies because of methodological differences. Varying prevalence rates have been reported in international studies like the Epidemiological Catchment Area Program and the National Comorbidity Survey.[5–6] Above all this, psychiatric disorders are known to vary across time within the same population and also vary across populations at the same time. This dynamic nature of the psychiatric illnesses will impact planning, funding and healthcare delivery. Providing accurate data about the prevalence of mental disorders is essential in policy-making. Hence this chapter attempts to critically evaluate the (overall) prevalence rate of psychiatric disorders as reported in epidemiological studies from India. In addition, it also attempts to address the following questions, a) What is the accurate prevalence of psychiatric disorders? b) Is the prevalence of psychiatric disorders reported in India similar to other international studies? c) Is the prevalence rate of psychiatric disorders stable or changing? d) Are there any population subgroups which are at a high risk of developing mental illness? e) What is the cost of treating psychiatric patients? and f) What should be the focus for future epidemiological studies?

### Methodology of the review

Extensive search was done on Neuromed (1982-1997), Pubmed, Indian Journal of Psychiatry website and MEDLAR for published Indian psychiatric epidemiological studies between 1960 and 2009. The search terms included, “Psychiatry” “Prevalence”, “Community”, and “Epidemiology”. Attempts were also made to retrieve Indian epidemiological studies published in international journals through Pubmed using the search terms, “psychiatry” and “epidemiology”. Extensive manual search of earlier and later issues of the Indian Journal of Psychiatry, NIMHANS Journal and ICMR journals was also done. Cross-references of the psychiatric prevalence studies were also reviewed by the authors.

Studies selected for the review were: General population studies, either urban, rural or mixed from India; community study design involving door-to-door/house-to-house enquiry of families and random sampling of families and inclusion of all psychiatric disorders or at least priority/major mental disorders (various researchers have used this term to assess schizophrenia, manic depressive psychosis, organic psychosis, epilepsy and mental retardation in the community). Studies were excluded if those were: Specific syndrome/illness/disorder studies and hospital or clinic-based studies. Only published data was considered due to logistic reasons. This is one of the main limitations of this review. Only 16 prevalence studies [Table 1] fulfilled the above cited criteria. However, the authors also made an attempt to summarize other studies like high-risk/special population, cost-effective analysis, incidence, follow-up and meta-analysis separately to have a comprehensive picture of epidemiological studies.

**Table 1:** Prevalence of psychiatric morbidity in the general population

Investigator	Year	Center	Location	Sampling	Tool	Population	Prevalence/1000
Surya[8]	1964	Pondicherry	U	H-H	MHSQ(P)	2731	9.5
Sethi *et al*.[9]	1967	Lucknow	U	H-H	QAPF	1733	72.7
Dube[10]	1970	Agra	M	H-H	DCP	29.468	18
Elnager *et al*.[11]	1971	Hoogly	R	H-H	CHM and DCP(2)	1393	27
Sethi *et al*.[12]	1972	Lucknow	R	H-H	CHQ and CHM	2691	39.4
Verghese *et al*.[13]	1973	Vellore	U	SRS	MHIS and DCP as per ICD (1965)	1887	66.5
Sethi *et al*.[14]	1974	Lucknow	R	3SPS	PSQ and DCP as per DSM-II (1968)	4481	67.0
Thacore *et al*.[15]	1975	Lucknow	U	H-H	PHQ and DCP	1977	81.6
Nandi *et al*.[16]	1975	West Bengal	R	H-H	HS, QS and CRS as per ICD (1965 R)	1060	102.8
Nandi *et al*.[17]	1979	West Bengal	R	H-H	HS, SESS, CDS and CRS	3718	102
Shah *et al*.[18]	1980	Ahmedabad	U	H-H	MHSQ and DCP	2712	47.2
Mehta *et al*.[19]	1985	Vellore	R	S-S	IPSS and DCP	5941	14.5
Sachdeva *et al*.[20]	1986	Faridkot	R	H-H	HS, SESS and CDS	1989	22.12
Premrajan *et al*.[21]	1993	Pondichery	U	RS	IPSS and DCP as per ICD-9R	1115	99.4
Shaji *et al*.[22]	1995	Erankulam	R	H-H	IPSS, SESS, CRS and DCP, ICD-10	5284	14.57
Sharma and Singh[23]	2001	Goa	M	SRS	RPES and DCP as per ICD-9	4022	60.2

Source: Math *et al*. 2007, IJMR, 183-192 Abbreviation - U - urban; R - rural; H-H - house to house survey; S-S.- systematic sampling; SRS - stratified random sampling 3SPS - 3-stage probability sampling; RS - random sampling, ICD - international classification of diseases DSM-II - diagnostic and statistical manual of mental disorders. Tools: MHSQ = Mental health screening questionnaire; QAPF = Questionnaire for the assessment of psychiatric state of the family; DCP = Diagnosis confirmed by a psychiatrist(s); CHM = Case history method; CHQ = Case history questionnaire; IPSS = Indian Psychiatric survey schedule; SFQ = Social functioning questionnaire; MHIS = Mental health item sheet; PSQ = Psychiatric screening questionnaire; PHQ = Psychiatric health questionnaire; HS = Household schedule; QS = Questionnaire schedule; CRS = Case record schedule; CDS = Case detection schedule; SESS = Socioeconomic status schedule; RPES = Rapid psychiatric examination schedule

### What is the prevalence of psychiatric disorders in India?

Prevalence can be simply defined as total number of persons in the population who have a psychiatric disorder at a point or period in time. It refers to both old (existing) and new (occurring) cases. In the above definition, if the observational period is at a given point in time it is called as ‘point prevalence’ and if it is at a given specific period in time it is called as ‘period prevalence’.[7]

Most of the community-based Indian epidemiological studies are on point prevalence. Table 1 summarizes the prevalence of psychiatric morbidity in the general population. These community-based epidemiological studies conducted in India on mental and behavioral disorders report varying prevalence rates, ranging from 9.5[8] to 102[16] per 1000 population.

### What are the reasons for wide variations in the prevalence of psychiatric disorders?

Prevalence rate of mental disorders vary within a population over a period of time and also across populations at the same time. This dynamic nature of mental disorders may play a role in the varying rates reported in Indian epidemiological studies. Similarly, on plotting the psychiatric epidemiological studies on the Indian map, studies are found to be concentrated only in certain places like [approximately] West Bengal (40%) and Uttar Pradesh (10%), which leads to difficulty in generalizing the findings.[3]

Defining a case is also one of the factors which contributed to the huge variation in the prevalence rate. If the threshold for defining a case is very low then the prevalence rate will be very high.[24–25] Mental disorders are highly stigmatized conditions that many people want to keep private because of embarrassment or fear of discrimination.[26] The problem of systematic under - reporting continues to be a major challenge for the future of psychiatric epidemiology in India. For example, a survey of an urban community in southern India found that one-third of people with schizophrenia had never accessed any treatment resources.[27] Even after the diseased individuals and their families were offered treatment, a third of them remained untreated.[28] In Indian epidemiological studies, many researchers interviewed only the head of the family or the housewife or any other responsible family member for data collection. This will lead to responder bias and also recall bias. There is a high chance of underreporting of symptoms of minor mental disorders [Table 2].[3]

**Table 2:** Reasons for wide variations reported in Indian epidemiological studies

Inherent nature of the psychiatric disorders Diagnostic methods Definition used to define a ‘case’ Systematic underreporting Recall bias Single informant Need for treatment Screening instrument Clinical interview, structured interview or semi-structured interview Sampling procedure Sampling bias

All the above factors played a crucial role in underreporting the prevalence rate in most of the Indian epidemiological studies. Because the screening instrument applied to the entire population had poor sensitivity in identifying minor mental disorders and also in high-risk populations such as the children and the elderly, it resulted in missing minor mental disorders during the initial screening. However, the majority of the researchers confirmed the diagnoses that were identified through the screening in the second phase avoiding false. positive cases. In addition to the poor screening instrument, recall bias, single informant and systematic underreporting have led to underreporting of mental disorders rather than over-reporting in Indian epidemiological studies

Though the majority of the epidemiological studies considered two-phase sampling for assessing prevalence,[8,10,14,15,18] they were unable to tap the non-psychotic disorders like panic disorder, social phobia, agoraphobia, adjustment disorder, dissociative disorder, obsessive compulsive disorder, sexual dysfunctions, substance use and so forth in the community. Earlier studies prepared their own screening questionnaires, which was applied to the entire population to be studied without testing their validity for high-risk populations such as children, elderly and substance users, resulting in missing out on the minor mental disorders during the initial screening.[8,11,14,15] This was a major drawback of these studies, which may have led to underreporting of mental disorders.

The sampling procedure employed should be appropriate, such that the sample obtained should be representative of the general population. If this is not achieved then the generalizability of the findings becomes difficult. Studies done on high-risk populations yielded a high prevalence rate. Hence, the selection should be representative of the general population. In the design of studies of people with mental disorders, in a community survey, it is also necessary to consider the likelihood of not being able to access people who are hospitalized due to illness, homeless people, wandering mentally ill patients and people who are not available for other reasons like occupation, custodial care, continuous care facilities and hospitalization for chronic physical illness.[29]

### What is the accurate prevalence of psychiatric disorders?

Policymakers are haunted by major discrepancies in the prevalence of mental disorders. Unfortunately, there is no sharp boundary between mental disorders and normalcy. Weak agreement at the level of diagnosis continues to threaten the credibility of estimates of prevalence.[30] However, efforts to overcome these discrepancies through meta-analysis and adding prevalence of individual disorders helped give meaning to the data, thereby enabling policy makers to plan service delivery.

#### Meta-analysis

A meta-analysis of 13 epidemiological studies consisting of 33,572 persons, who met the following criteria: Door to door survey, all age groups included and prevalence rate for urban and rural being available,[31] reported a total morbidity of 58.2 per 1000. Though meta-analysis has its own limitations, this was the first attempt to analyze the epidemiological studies. Another meta-analysis of epidemiological studies reported a total morbidity of 73 per 1000.[32] The difference noted in the two available meta-analyses is due to the methodology of selecting the papers for the review [Table 3].

**Table 3:** Meta-analysis of Indian epidemiological studies

Investigator	Year	Population	Prevalence/1000
Reddy and Chandrasekar[31]	1998	33,572	58.2
Ganguli[32]	2000	-	73

Source: Math *et al*., 2007, IJMR, 183-192

#### Macro-economic commission report

Macro-economic commission report of 2005[33] considered prevalence rate of 65/1000 population (average of two meta-analyses) and projected the prevalence rate for the next two decades [for more details please see the report of NCMH Background Papers - Burden of Disease in India[33]].

#### Modest estimation of Psychiatric Morbidity

If we consider the prevalence of individual mental disorder and add, then the overall prevalence rate is approximately 190-200/1000 population [Table 4]. In simple words, at least 20% of the population does have one or the other mental health problem, which requires intervention from a mental health professional. This estimate has been developed based on secondary data from available sources along with an in-depth review of existing databases in the Indian region. Only core psychiatric disorders have been addressed in this estimate. Considering the fact of systematic underreporting, collecting data from single informant, use of low-sensitivity screening instruments and assessing only priority mental disorders, the prevalence of mental disorders reported in Indian epidemiological surveys can be considered *lower estimates* rather than accurate reflections of the true prevalence in the population.[3]

**Table 4:** Modest estimate of the mental health morbidity

Mental Disorders	Prevalence/1000	%	Total population in crores*	Mental morbidity in lakhs
Schizophrenia and other psychotic disorders (including organic)	5-10	1	108	108
Mood disorders	15-30	3		324
Cannabis users	5-10	1		108
Opiate users	1-3	0.3		32
Mental retardation	5-6	0.6		65
Dementia	2-5	0.5		54
Common mental disorders	20-30	3		324
Alcohol dependence syndrome	30-40	4		432
Child and adolescent disorders	110-120	12	40	480
Geriatric disorders	25-30	3	8	24
				1951

* As per census 2001 above table does not include the following psychiatric morbidities: One-third of the patients suffering from any chronic medical conditions (such as Diabetes, Hypertension, Cancer, Asthma, Ischemic heart diseases, Arthritis, HIV, Psoriasis, Chronic renal failure, Cerebro-Vascular accidents, Epilepsy, Auto-immune disorders, Obesity, infertility and so forth) also have co-morbid diagnosable psychiatric disorders, which are usually not diagnosed and never treated. Similarly, one-third of the patients attending the outpatient section of any primary health center or general hospitals suffer from diagnosable psychiatric disorders such as psychosomatic disorders, somato form disorders, medically unexplained symptoms, depression, anxiety disorders, sleep disorders, sexual dysfunction, premenstrual syndrome and so forth

### Is the prevalence of psychiatric disorders reported in India similar to other international studies?

#### Comparison with International studies

NIMH-Epidemiological Catchment Area study[34] of the US reported psychiatric morbidity as follows; one year incidence of 60/1000 population, one month prevalence of 151/1000 population and lifetime prevalence of 322/1000 population. National Co-morbidity Study of the US[35] reported 12 months prevalence of 277/1000 population and lifetime prevalence of 487/1000 population. On comparing the Indian epidemiological studies to any international epidemiological studies, it is found that prevalence rates reported in India are very low. Possible reasons for this difference have been discussed in Table 5.

**Table 5:** Low prevalence of psychiatric disorders in India can be attributed to the following reasons

Indian epidemiological studies were not able to measure psychiatric morbidity adequately
Psychiatric prevalence rates are truly low in India because of
Genetic reasons,
Good family support,
Social support,
Cultural Factors
Lifestyle
Better coping skills and comfortable environment
Combination of above factors

Available evidence supports the first possibility of the underreporting by Indian epidemiological studies. However, the remote possibility of genuine low prevalence of psychiatric disorders in the Indian population cannot be disregarded because of low rates of substance use in the general population compared to Western countries and good outcome of psychiatric disorders due to various factors like better coping skills, religious, cultural, social and family support.

### Is the prevalence rate of psychiatric disorders stable or changing?

Does globalization and recession play a role in the mental health of individuals? Does changing family structure (joint family to nuclear) influence psychiatric morbidity? Unfortunately, we don’t have any long-term epidemiological studies to answer these questions.

#### Follow-up studies

A 10-year follow-up study reported that there was not much change in the prevalence rates over a decade.[36] This was further reinforced by the 20-year follow-up study.[37] These two studies [Table 6] are milestones in psychiatric epidemiology that focus on the same population cross-sectionally at two points. Though the prevalence rate did not change, the morbidity pattern changed significantly. However, researchers and policymakers should exercise caution before generalizing the findings to the entire country. Reason being, follow-up studies had a small sample size and were done in a rural population. Researchers should also consider the changing trend in the Indian population before generalizing the findings.

**Table 6:** Follow-up studies on the prevalence of psychiatric disorders in India

Investigator	Year	Center	Location	Sampling	Tool	Year	Population	Prevalence/1000
Nandi *et al*.[36] (1972-1982)	1986	West Bengal	R	H-H	HS, SESS, CDS and CRS	1972	1060	84.9
						1982	1539	81.9
Nandi *et al*.[37] (1972-1992)	2000	West Bengal	R	H-H	HS, SESS, CDS, CRS and DCP	1972	2183	116.8
						1992	3488	105.2

As given in the footnote of Table 1; Source: Math *et al*., 2007, IJMR, 183-192

#### Incidence studies

There are only two incidence studies [Table 7] conducted in India,[38–39] there is a need to carry out more incidence studies in representative populations. Incidence studies are essential to know the impact of intervention programs, globalization, recession, disaster and so forth.

**Table 7:** Incidence studies done in India

Investigator	Year	Center	Location	Sampling	Tool	Year	Population	Prevalence/1000	Incidence/1000
Nandi *et al*.[38] (1972-1973)	1976	West Bengal	R	H-H	HS, QS and CDS	1972	1060	102.1	17.6
						1973	1078	107.6	
Nandi *et al*.[39] (1972-1973)	1978	West Bengal	R	H-H	HS, CDS and CRS	1972	2230	110.3	16
						1973	2250	108.4	

As given in the footnote of Table 1; Source: Math *et al*., 2007, IJMR, 183-192

Though there are no systematic studies in answering the above questions. However, an increase in population has definitely increased the number of mentally ill patients in India. Lack of mental health manpower is a major threat to developing comprehensive psychiatric services in the community. In spite of best efforts, the ratio between psychiatrist and population is worsening day-by-day. Main reason being, the Indian population is growing at a rapid speed while the development of manpower is not. There are no attempts to address the issue of manpower in the area of mental health.

### Are there any population subgroups which are at high risk of developing mental illness?

Various studies have clearly shown that the prevalence of psychiatric disorders is high in certain population. Population at high risk of develop psychiatric disorders are depicted in Table 8.

**Table 8:** Population at high risk of develop psychiatric disorders are as follows

Female gender
Child and adolescent population
Students
Geriatric population
People suffering from chronic medical conditions
Disabled population
Disaster survivors
Population in custodial care
Marginalized population
Refugees and individuals with poor family, social and economical support

### Prevalence of psychiatric disorders in child and adolescent population studies

Children and adolescents are at high risk of developing mental disorders. The majority of available Indian general population prevalence surveys [Table 1] have not utilized specific tools for addressing the disorders in children and adolescents. They have formulated their own screening instruments or they have utilized screening instruments which can be applied to the adult population, hence no doubt they have missed out mental morbidity in children and adolescents. A review article by Bhola and Kapur[40] has summarized the prevalence of psychiatric disorders in the child and adolescent population. They have reviewed both community and also school-based population studies. Early studies reported prevalence rates of psychiatric disorders among children ranging from 13 to 94 per 1000.[16,41–42]

There are only a few epidemiological studies which were exclusively conducted to assess the prevalence rate in the child and adolescent population. The first methodologically superior study reported a prevalence rate of 94 per 1000 in a sample of 1403 rural children aged 8-12 years.[43] Another methodologically strong ICMR-sponsored study conducted by Srinath and colleagues in 2005, has reported a prevalence rate of 12.5% among children aged 0-16 years.[44] Similarly, a recent study also reported a prevalence rate of 16.5% in children 6-14 years of age.[45] Table 9 summarizes the prevalence rate of the child and adolescent population. Since children and adolescent form 40% of the total population of India,[46] approximately four crores of the population require professional help.

**Table 9:** Prevalence of psychiatric morbidity in child and adolescent population studies

Investigator	Year	Center	Age (yrs)	Location	Sampling	Tools	Population	Prevalence/1000
Sethi *et al*.[9]	1967	Lucknow	0-10	U	H-H	IQ, ICD-7	541	94
Dube *et al*.[10]	1971	Agra	5-14	M	H-H	DCP	8035	11.67
Elnagar *et al*.[11]	1971	Hoogly	0-15	R	H-H	CHM and DCP(2)	635	13
Sethi *et al*.[12]	1972	Lucknow	0-10	R	H-H	IQ, ICD-7	877	81
Varghese *et al*.[42]	1974	Vellore	4-12	U	SRS	MHIS and DCP as ICD (1965)	747	81.7
Nandi[16]	1975	Kolkata	0-11	R	H-H	IQ, DCP as per ICD (1965)	462	26
Hackett *et al*.[43]	1999	Kerala	8-12	U	RCS	CBQ, ICD-10	1403	94
Srinath *et al*.[44]	2005	Bangalore	0-16	U	SMS	SDP, SCL, CBCL, CBQ, FTN, DISC, PIS, VSMS, BKT, CGAS	2000	124
Anita *et al*.[45]	2007	Rohtak	6-14	M	SRS	CPMS and DISC	800	165

U - urban; R - rural; M - Mixed; H-H - house to house survey; RCS - Random cluster sampling; SRS - stratified random sampling; SMS - stratified multistage sampling; ICD - international classification of diseases; Tools - IQ - Interview questionnaire; CHM - Case history method; DCP - Diagnosis confirmed by a psychiatrist(s); MHIS - Mental health item sheet; CBQ - Child behavior questionnaire; SCL - Screening checklist; SDP - Socio demographic proforma; CBCL - Child Behavior; Checklist PIS - Parent interview schedule; BKT - Bitnet karat test; VSMS - Vineland social maturity scale; FTN - Felt treatment needs CGAS - Children�s global assessment scale; DISC - Diagnostic interview schedule for children; CPMS - Childhood Psychopathology Measurement Schedule

#### Geriatric population

Senior citizens are at a high risk of developing mental disorders. The geriatric population, aged 60 years and above, forms nearly 7.5% of the total population of India.[46] A study conducted in two villages of West Bengal reported that 61% of the geriatric population needed psychiatric help.[47] Majority of them were suffering from depression. Other commonly reported mental disorders are insomnia, sexual dysfunction, anxiety disorders, somatoform disorders, organic mental disorders and dementia. Depression is a common cause of disability in the elderly. Consequences of untreated depression are, reduced life satisfaction and quality, social deprivation, loneliness, increased use of medications and health services, insomnia, cognitive decline, suicide and increased mortality. There is a definite need for conducting systematic studies in the geriatric population to estimate the prevalence of psychiatric disorders.

#### Disaster and mental health

Disasters are potentially traumatic events which impose ‘massive collective stress’ consequent to ‘violent encounters with nature, technology or mankind’.[48] Disasters threaten personal safety, overwhelm defense mechanisms, and disrupt community and family structures. They may also cause mass casualties, destruction of property, and lead to a collapse of the social networks and daily routines.[49] A typical pattern of mental, emotional, and physical response is observed in the majority of people after exposure to any disaster.[50] Various international studies have reported a wide range from 30-70% of mental health morbidity as an immediate aftermath of a disaster. A meta-analysis of 160 international studies of disaster victims found that posttraumatic stress disorder, major depressive disorder, generalized anxiety disorders and panic disorder, were commonly identified by most of the studies.[51] The disaster mental health branch lags behind in terms of coordination, training, services, research and evidenced-based practices in India.

#### Other high-risk populations

In Indian epidemiological studies researchers have sampled special population groups [Table 10] like urban slum dwellers,[52] uprooted communities,[53] urbanized tribal communities[54–55] and attempted to compare across cultures.[56–57] Studies done on the high-risk population yielded high prevalence rates.[24]

**Table 10:** Special/high-risk population studies

Investigator group	Year Center	Nature of risk	Location	Sampling	Tool	Population	Prevalence/1000
Carstairs and Kapur[24]	1973	Social changes in the community	R	H-H	IPSS and SFQ	1233	370
Nandi *et al*.[56]	1977	Tribal community	R	H-H	HS, QS and CDS	2918	58.2
Nandi *et al*.[53]	1978	Uprooted community	R	H-H	HS, QS and CDS	1259	47.6
Nandi *et al*.[57]	1980	Marginalized population	R	RS	HS, SESS, CDS and CRS	4053	50.3
Nandi *et al*.[58]	1980	Urbanization	M	H-H	HS, SESS, CDS and CRS	1862	129.9
Sen *et al*.[52]	1984	Urban slum dwellers	U	H-H	HS, SESS, CDS and CRS	2168	48.7
Banerjee *et al*.[54]	1986	Urbanized tribal community	U	H-H	HS, SESS, CDS and CRS	771	51.9
Nandi *et al*.[55]	1992	Urbanized tribal community	U	H-H	HS, SESS, CDS and CRS	1424	47.75

As given in the footnote of Table I Source: Math *et al*., 2007, IJMR, 183-192

### What is the economic cost of treating psychiatric patients?

Prevalence of mental illness is approximately 200/1000 population [Table 4]. In simple words, approximately 20 crores of the population require professional help. Each mentally ill patient requires Rs. 500 per month for mental healthcare [Table 11]. This includes medication cost, doctor’s fees and travelling cost to meet the doctor. Then the approximate total cost required per month will be Rs.10,000 crores. Unfortunately, mental illness requires medication for longer duration. Most of them require medications ranging from several months to years. Many of these disorders if not detected early and treated, may become chronic and require medication for life. If we don’t address this issue, then the indirect costs in terms of loss of wages, disability, absenteeism and substance use is unimaginable. Above all these families encounter social isolation, burden, stigma, poor quality of life and enormous psychological strain.

**Table 11:** Cost of treating mentally ill patients

	Per month (Rs.)
Cost for mental healthcare for an individual	
Medication cost per month for an individual suffering from mental illness	300
Traveling cost to meet the mental health professionals	100
Doctors fees (mental health professionals)	100
Total	500
Cost for mental healthcare for the whole country	
If we consider the psychiatric prevalence as 200/1000 population (see table 4), then 20 crores of the population require professional help. (Prevalence is 20 crore population X Cost per month per patient is 500 Rs)	10,000 crores

### Is there a need to fund psychiatric epidemiological studies?

The new generation of mental health surveys required at this point of time are to determine the quality of life, co-morbidity, disability and burden of various mental disorders.[59] There is a need for longitudinal/prospective (experimental) epidemiological studies in which the natural course of all the disorders in the community can be studied and modifiable risk factors identified and targeted for interventions. It is also vital to study the factors (barriers) affecting better service use, the role of culture and religion in help-seeking behaviors and modifying the identified factors, which may help in better delivery of mental healthcare at the community level. Mental healthcare priority also requires to be shifted from mental hospitals to primary health centers.[60]

Epidemiological studies are expensive and time-consuming. Sponsoring agencies have to be sensitized to contribute to the area of psychiatric epidemiology. Above all this, the scarce availability of resources like manpower, funding, time, practical difficulties in the field and so forth have led researchers to think many times before embarking on psychiatric epidemiological studies, which explains the number of publications in the past ten years. Without addressing these issues we may not be able to move further in this field.[61]

## CONCLUSIONS

Mental health problems constitute a wide spectrum ranging from sub-clinical states to very severe forms of disorders. Majority of the epidemiological studies focused on visible mental health problems. Invisible mental health problems continue to remain unexplored and unaddressed. Mental healthcare priorities need to be shifted from psychotic disorders to common mental disorders and from mental hospitals to primary health centers.

Indian psychiatric epidemiological researchers had taken the herculean task of bringing the numbers to the policymakers since 1964. This is truly commendable considering the challenges faced, such as meager human and financial resources. However, their endeavor does not seem to have received the attention that it richly deserves in the national and international arena. Nevertheless it is still not too late to leverage the findings and invest in mental health. Available evidence indicates the overall prevalence rate is approximately 190-200/1000 population. In simple words, at least 20% of the population does have one or the other mental disorder, which requires the mental health professionals’ intervention. This needs to be considered as a modest estimation of the psychiatric prevalence in the Indian population, for policy making. It is high time to stop the long-term debate about the prevalence rate of mental illness in India and move forward to actual actions that call for investing and improving the mental health services in India.

The need of the hour is in addressing major challenges such as lack of mental health manpower, financial aid and stigma, which are the major threats to developing comprehensive psychiatric services in the community. In spite of best efforts, the ratio between psychiatrist and population is worsening day-by-day. Feeble attempts to address the issue of development of manpower in the area of mental health are far from reality. Adding to this, natural and manmade disasters are also placing an enormous challenge on the available meager resources. Increase in invisible mental health problems such as suicidal attempts, aggression and violence, widespread use of tobacco, alcohol and other drugs, increasing marital discord and divorce rates emphasize the need to prioritize and make a paradigm shift in the strategies to promote and provide appropriate mental health services.
